# Value of Micro-CT for Monitoring Spinal Microvascular Changes after Chronic Spinal Cord Compression

**DOI:** 10.3390/ijms150712061

**Published:** 2014-07-07

**Authors:** Hou-Qing Long, Wen-Han Xie, Wen-Li Chen, Wen-Lin Xie, Jing-Hui Xu, Yong Hu

**Affiliations:** 1 Department of Spine Surgery, the First Affiliated Hospital of Sun Yat-Sen University, Guangzhou 510000, China; E-Mails: xiewenh@mail2.sysu.edu.cn (W.-H.X.); xjhbest1818188@163.com (J.-H.X.); 2 Department of Neurosurgery, the First Affiliated Hospital of Sun Yat-Sen University, Guangzhou 510000, China; E-Mail: chenwenli06@163.com; 3 Department of Pathology, the First Affiliated Hospital of Sun Yat-Sen University, Guangzhou 510000, China; E-Mail: xiewenlin.55@163.com; 4 Department of Orthopaedics and Traumatology, Li Kai Shing Faculty of Medicine, the University of Hong Kong, Pokfulam, Hong Kong, China

**Keywords:** chronic spinal cord compression, micro-computer tomography, immunohistochemistry, somatosensory evoked potential, blood vessels

## Abstract

Neurological degeneration can occur after compression of the spinal cord. It is widely accepted that spinal cord compression leads to ischemic lesions and ultimately neurological dysfunction due to a narrowed spinal canal. Therefore, an in-depth understanding of the pathogenesis of spinal cord compression injury is required to help develop effective clinical interventions. In the present study, we propose a new method of quantitative 3D micro-CT to observe microvascular events in a chronic spinal cord compression rat model. A total of 36 rats were divided into two groups: sham control group (*n* = 12) and compressive spinal cord injury group (*n* = 24). Rats were scarified at four weeks after surgery. In each group, CD34 micro-vessel immunohistochemical staining was performed in half of the animals, while micro-CT scanning was performed in the other half. Microvessel density (MVD) was measured after immunohistochemical staining, while the vascular index (VI) was measured in 3D micro-CT. In comparison with sham control, abnormal somatosensory evoked potentials (SEP) can be seen in all 24 cases of the compression group, and VI shows the amount of microvessels reduced consistently and significantly (*p* < 0.01). A significant correlation is also found between MVD and VI (*r* = 0.95, *p* < 0.01). These data suggest that quantitative 3D micro-CT is a sensitive and promising tool for investigating microvascular changes during chronic compressive spinal cord injury.

## 1. Introduction

Chronic cervical spinal cord compression is a common pathological scenario. For example, cervical disc herniation [1], ossification of the posterior longitudinal ligament (OPLL) [2] and intraspinal tumors [3] can lead to chronic cervical spinal cord compression, which will cause neurological dysfunction and seriously affect patients’ quality of life [4]. Previous studies have determined that spinal cord ischemia induced by compression is one of the predominant pathogenic mechanisms of this neurological dysfunction. For example, chronic ischemia and hypoxia to the spinal cord induced by impaired microvasculature can cause glial cell and neuronal injury [5].

Widely used traditional methods for detecting microvascular changes in the spinal cord include serial tissue sectioning [6], immunohistochemical staining of blood vessels [7] and thick tissue section angiography methods [8]. However, disadvantages of these techniques include cumbersome procedures, inability to perform imaging of the entire spinal microvascular network and inaccurate quantitative analysis. Microcomputer tomography (micro-CT) [9] is a three-dimensional imaging technique that can provide a clear understanding of internal tissue microstructure. A previous study proposed a method using *in vivo* micro-CT to monitor pathological changes of neurodegeneration in the brain [10]. To further develop a 3D image method for assessing microvascular architecture in the spinal cord, the usefulness of micro-CT in the monitoring of pathological changes during spinal cord injury (SCI) should be verified.

In this study, we compared the efficacy of three-dimensional micro-CT imaging *versus* CD34 immunohistochemical study for quantitatively assessing spinal microvascular changes in a rat model of chronic spinal cord compression.

## 2. Results

### 2.1. Basso Beattie Bresnahan (BBB) Score and Somatosensory Evoked Potentials (SEP)

**Figure 1:**
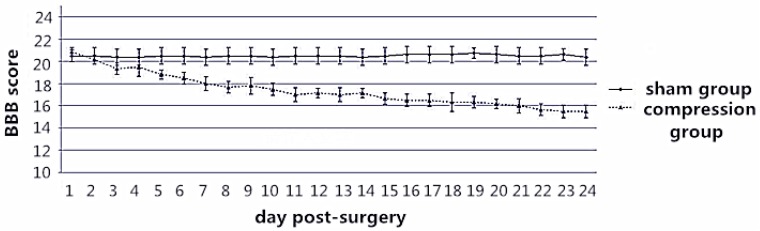
Basso Beattie Bresnahan (BBB) score continued to decline in the compression group, while there was no significant change in the sham group.

According to the BBB score, neurological function in the compression group showed a marked decline from one to two weeks after surgery, and a smooth decrease from two to four weeks after surgery (Figure 1, Table 1, *p* < 0.05). BBB score was significantly lower in the compression group compared with that in the sham control group.

**Table 1:** Average BBB score of the two groups in checking compression time (* *p* < 0.05).

Group	4th Day	12th Day	24th Day
Sham (*n* = 12)	20.87 ± 0.12	20.82 ± 0.11	20.89 ± 0.14
Compression (*n* = 24)	18.74 ± 0.21 *	16.63 ± 0.19 *	15.22 ± 0.18 *

**Figure 2:**
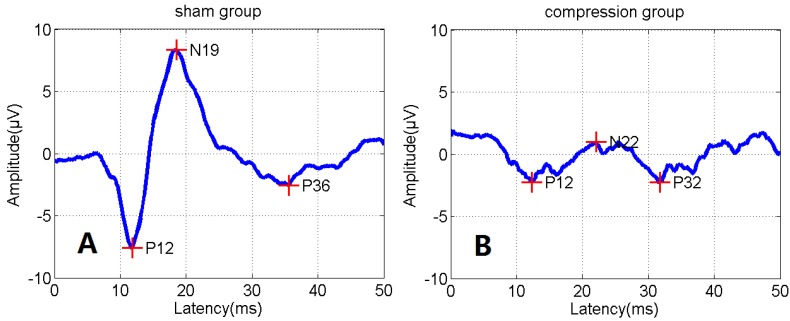
A sample of latency prolongation and amplitude reduction of somatosensory evoked potentials (SEP) from normal status (**A**) to compression status (**B**).

In addition to BBB assessment, SEP tests were performed in all groups to evaluate functional changes after SCI [11,12]. Amplitude in the compression group presented a significant reduction, while latency presented a significant prolongation compared to the sham group (Figure 2, Table 2, *p* < 0.05).

**Table 2:** Average amplitude and latency of the two groups (* *p* < 0.05).

Group	Latency (ms)	Amplitude (μV)
Sham (*n* = 12)	4.26 ± 0.21	7.17 ± 0.12
Compression (*n* = 24)	8.47 ± 0.31 *	3.22 ± 0.21 *

### 2.2. CD34 Semi-Quantitative Analysis

**Figure 3:**
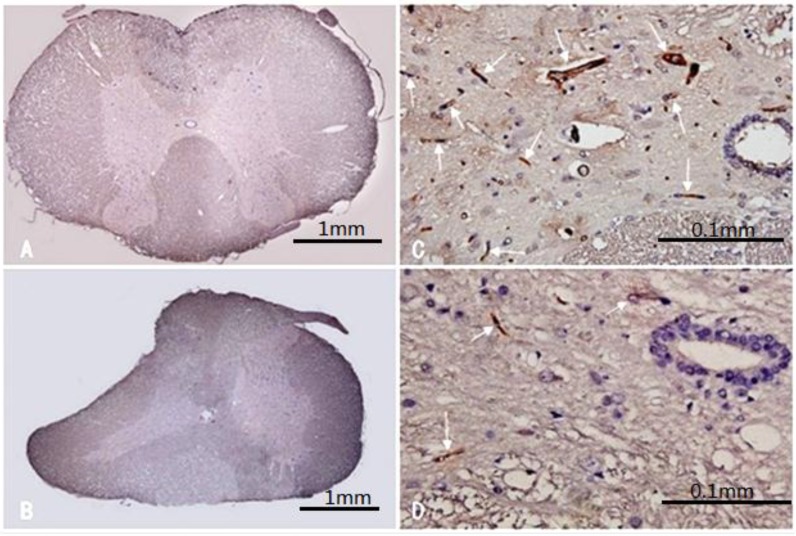
CD34 immunohistochemical staining in normal and compressed spinal cord segment. (**A**) Normal spinal cord contour in control group (×4); (**B**) CD34-positive vascular endothelial cells sparsely distributed in the gray matter at high magnification in control group (×40); (**C**) deformedspinal cord contour in compressed group (×4); (**D**) compression spinal cord section at high magnification, microvascular density in gray matter decreased (×40). “→” shows the CD34-positivecapillary.

**Figure 4:**
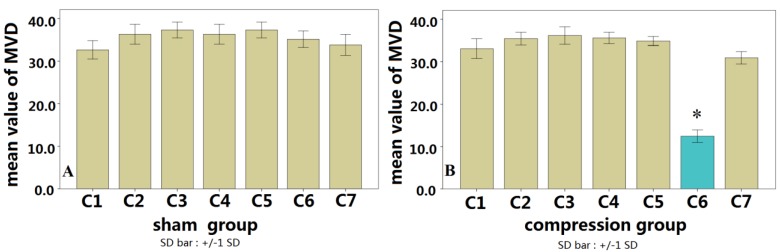
No difference among each segment in the sham group regarding microvessel density (MVD) (**A**); while the mean value of MVD in C6 of the compression group showed significantly lower than that in C6 of the sham group (* *p* < 0.01) (**B**).

The spinal cord sections in the sham control group displayed a normal contour at low magnification (Figure 3A). At high magnification, there were numerous CD34-positive vascular endothelial cells [13]. The microvascular system was also clearly outlined (Figure 3B). In the compression group, the contour of the spinal cord was deformed (Figure 3C), the lumens of the microvascular system in compressed spinal cord gray-matter regions were narrowed, and the numbers of microvessels in gray-matter were significantly decreased (Figure 3D). The overall numbers of microvessels assessed by MVD in the compression segments was decreased significantly compared with that of the normal segment in the sham control group (*p* < 0.01, Figure 4).

### 2.3. Micro-CT Detection

**Figure 5:**
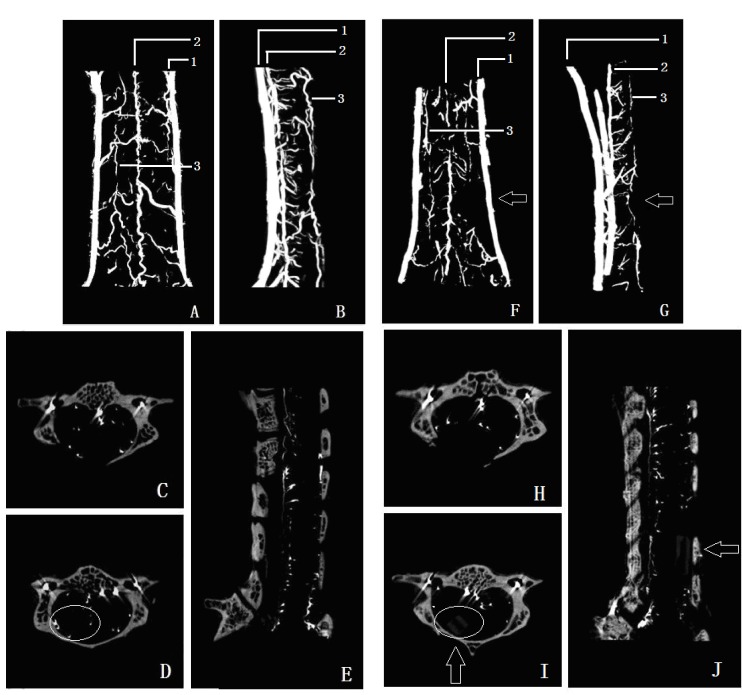
3D microvessels reconstruction image of sham group on coronal and lateral view (**A**,**B**); cross sectionof C5 and C6 in sham group (**C**,**D**); sagittal view of sham group (**E**); 3D microvessels reconstruction image of compression group on coronal and lateral view (**F**,**G**); arrow shows the microvessels decreasing region; cross section of C5 and C6 in compression group (**H**,**I**); sagittal view of compression group (**J**); circle shows the difference microvascular index in C6 between sham and compression group (**D**,**I**).

As shown in Figure 5, the contrast micro-CT of the microvascular system was presented in 3D coronal view (Figure 5A,F), in 3D sagittal view (Figure 5B,G), in 2D sagittal view (Figure 5E,J), and in cross section view (Figure 5C,D,H,J).To compare the microvascular change in the compression area, a 2D cross section in the compressor inserting site and the compression site were shown in Figure 5H,I, 2D sagittal section in sham group and compression group were showed in Figure 5E,J. In 3D micro-CT imaging, the vertebral artery (marker 1 in Figure 5A,B,F,G), anterior spinal artery (marker 2 in Figure 5A,B,F,G) and the arteria spinalis posterior (marker 3 in Figure 5A,B,F,G) can be clearly seen.

**Figure 6:**
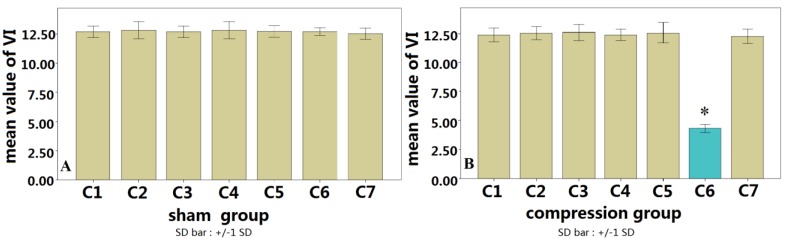
No difference among spinal levels C1–C7 in the sham group in terms of VI (**A**); while the mean value of VI at spinal cord C6 of the compression group showed significantly lower than that in C6 of the sham group (* *p* < 0.01) (**B**).

The compression sheet was observed in the micro-CT imaging at C6 level (Figure 5E,J). After compression to the spinal cord, the course of the anterior spinal artery was disrupted and moved forward in the compression segment (Figure 5G). The branches were absent in the posterior and lateral funiculus of the spinal cord (Figure 5D,I), the quantity of microvessels decreased significantly compared with that in the sham control group (Figure 5A,F and Figure 6).

### 2.4. Correlation Analysis

**Figure 7:**
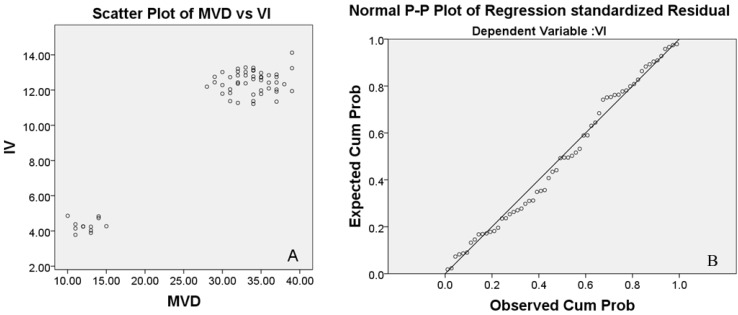
*r* = 0.95，*p* = 0.001, GFI = 0.903, Correlation analysis of the two methods were significantly associated at the level of 0.01 (double side, **A**); Regression index analysis: *b* = 0.352, *p* = 0.001 for additional 1 in MVD, VI will increase 0.352, *t*-test was passed at level of 0.05 (double side, **B**).

Correlation analysis showed that micro-CT detection was significantly associated with CD34 semi-quantitative analysis (Figure 7).

## 3. Discussion

The pathogenesis of chronic compressive spinal cord injury (myelopathy) remains to be fully understood. Animal experiments and human autopsy studies suggested that compression of arterial blood supply to the spinal cord is the most likely cause of spinal cord ischemia [14,15,16]. Accordingly, in the present study, we focused to investigate the intramedullary microcirculation changes after chronic compression of the spinal cord. 

A number of techniques for visualizing spinal cord microvasculature have been reported, including spinal cord transverse section continuous biopsy, immunohistochemical staining of blood vessels, and angiography [6,7,8]. Continuous histology sectioning is a classical method to obtain histological information [6]; however, it requires hundreds of sections in order to obtain vascular morphology information. At the same time, errors can often occur during serial sectioning, which can affect the reliability of histological observation. Immunological staining of vascular endothelial cells [7] can provide vessel information using optical or fluorescence microscopy, which can provide information on pathological changes in blood vessels, reflecting the function of the vascular component. However, 3D images to observe the whole system of the blood vessel volume cannot be obtained. Angiography [8] can provide whole capillary images for examining the spinal cord microvasculature. However, it is difficult to image the tiny microvessels because of the limitation of the X-ray source. Furthermore, the 2–3 mm thick spinal radiography produces an image of multiple overlapping microvessels, which lacks specificity for lesion area identification. As such, more sensitive and specific microvessel detection methods are required.

Micro-CT has been used for imaging teeth, placental blood vessels, blood vessels of organ tumor, and bone structure [17,18,19]. The microvascular resolution of the micro-CT system used in the present study was up to 10 μm, which allows detailed spinal cord microvascular to be detected. More importantly, microvascular volume information can be obtained by the 3D reconstructions, which is important for studying the overall change in microvascular volume. In the present study, we found that the posterior spinal artery was compressed and moved forward after spinal cord compression. Using the 3D micro-CT data, the density of microvascular supply from the anterior spinal artery in the compression segment spinal cord was reduced compared with that of a normal segment, indicating that reduced blood supply in the anterior spinal artery can lead to a decrease in microvascular density. In contrast to other studies, we used lead oxide-gelatin as the contrast agent, as its diameter is less than that of an arteriole, but larger than a capillary. Accordingly, the arterial blood-supply systemof the spinal cord can be displayed independently, which avoids the interference due to venous system filling.

According to anatomy, approximately 60%–70% of the spinal cord blood supply comes from the anterior spinal artery, and its terminal branch was the *rami anastomoticus* with the anterior spinal artery. The anterior spinal artery is sensitive to ischemia after chronic spinal cord compression [20]. Shimomura *et al*. [16] established an anterior spinal artery occlusion model of ischemia in rats, and demonstrated that only a third of the area of the ventral spinal cord was damaged by blocking anterior spinal artery blood flow, while the pyramidal tract was not involved. These results differ from the typical features of cervical spondylotic myelopathy (CSM) patients, where the classic clinical symptoms of CSM involve pyramidal tract damage syndrome caused by chronic spinal cord compression. Accordingly, injury to the spinal cord surface and intramedullary microvessels is likely to be an important component of the pathogenesis of chronic spinal cord compression myelopathy, while the anterior spinal artery is an important source of blood supply for the spinal cord. 

The chronic compression myelopathy rat model that we developed involved compression from the posterior end of the spinal cord, which is similar to the clinical scenario of CSM [11,12]. Micro-CT 3D images showed that the microvascular supply from the anterior spinal artery was significantly reduced. The BBB score and SEP results also suggest that the reduction of blood supply in the anterior spinal artery system can induce neurological dysfunction, which is similar to the studies of Shimomura *et al*. [16]. We also found that the microvascular quantity of the compressed segmental spinal cord was significantly reduced compared with that of the normal segment. These data were confirmed by histological and histochemical results, which indicate endothelial cell staining on spinal cord transverse sections at five weeks after spinal cord compression, as established by Karadimas *et al*. [21]. An alternative CSM rat model developed by Kurokawa [14] used a fluorescent dye that could not pass through the blood vessel wall. The compressed segmental spinal cord tissue was then extracted and detected using fluorescent spectrophotometer quantitative analysis after four weeks recovery. In that study, the fluorescent dye content in the compression group was decreased compared with the controls, and segmental cord blood flow was significantly reduced. In consistent with the findings in Kurokawa’s study [14], 3D micro-CT images confirmed that the density of spinal cord capillaries is decreased in the compressed segment, suggesting that the reduction in blood supply in the anterior spinal artery system led to decreased microvascular density. Another paper reported that the microvascular density was decreased in early stage after contusion injury to the spinal cord, then gradually increased in 7 to 14 days after injury [22]. In this study, the decreasing microvascular density was also observed in early stage of chronic compressive spinal cord injury. During chronic compression to the cord, there is no acute mechanical insult [23]. The change in architecture of spinal cord microvasculature may show a different pattern from acute SCI. It should be considered to observe the microvessel intensity in a longer period in further study.

We observed a significant correlation between the vascular index (VI) by CD34 immunohistochemistry and microvessel density (MVD) measured by micro-CT quantification. CD34 immunohistochemistry (MVD) and the micro-CT quantification (VI) were consistently reduced in the compressed spinal cords, confirming the feasibility of using micro-CT for the detection of spinal cord microvascular changes. Correlation analysis showed a significant correlation between MVD and VI, indicating that micro-CT has the same accuracy and sensitivity to detect microvascular architecture in the spinal cord as traditional immunohistochemical methods.

The merit of using micro-CT is to provide 3D image and the possibility of quantitative measurement of internal tissue microstructure, which is a difficulty in histological investigations. A limitation of this study, however, is that micro-CT was used here *in vitro*. A previous study has shown that micro-CT can, in principle, be done *in vivo* [10]. With the knowledge of the capability of the micro-CT to monitor the spinal cord vasculature status, it encourages us to proceed to develop an *in vivo* protocol for micro-CT. To address this issue, we will have to develop a new contrast-medium with lower stickiness and an intravenous injection device that allows a precise speed control of injection. The *in vivo* micro-CT may provide a new method to monitor pathological changes during chronic compressive spinal cord injury. In addition, various pathological alterations, *i.e*., reactive gliosis, neuronal necrosis, neovascularization, could be investigated during the progression of chronic spinal cord compressive injury in tissue won following *in vivo* micro-CT analyses to better correlate imaging results to tissue based events.

## 4. Experimental Section

The experiment procedure was approved by the Research Ethics Committee of the authors’ institute. A total of 36 adult Sprague-Dawley (SD) rats (280–300 g) were allocated to two groups: sham control group (*n* = 12) with immunohistochemical (*n* = 6) and micro-CT study (*n* = 6); compressive spinal cord injury group (*n* = 24) with immunohistochemical (*n* = 12) and micro-CT study (*n* = 12). In the compressive group, all rats were anesthetized with 10% chloral hydrate (3 mL/kg), the C5 lamina was exposed. Then the ligamentum flavum and partial lamina were removed to access the epidural space, and a compression sheet was implanted into the C6 epidural space on the posterolateral side to induce a compression to the cord. The sustained-release membrane was made of polyurethane, which was synthesized in the laboratory by isocyanates and polyols (Guangzhou Fischer Chemical Co., Ltd., Guangzhou, China). This compression material did not show any inflammatory reaction or tissue granulation after implantation in previous studies [12].

After complete hemostasis, the incision was closed by layers. For rats in the sham control group, the C5 laminae were removed without inserting the compression sheet. All animals were given an intramuscular injection of Penicillin G (8000 U/100 g, intramuscular injection) to prevent infection during the surgery. After surgery, animals were housed in cages individually and allowed free access to food and water. Post-operative analgesia was applied to rats with subcutaneous injection of buprenorphine (0.01 mg/kg) 12 hourly for 3 days. After 3 days, analgesia would be applied when the rat was observed any signs of pain or distress. In this study, no rat received analgesia after 3 days.

### 4.1. Motor Function and Neurophysiological Monitoring

To ensure effective compression, severity of paralysis due to spinal cord compression was evaluated in terms of motor function by using a BBB score. For all the rats, BBB scores were evaluated once a day from 24 h to 4 weeks post-surgery. Each rat received an SEP test [11,12] before euthanasia.

### 4.2. Specimens and Immunohistochemistry

At four weeks post-surgery, the cervical spinal cord from C3–C7 were processed for CD34 immunohistochemical staining for microvessel density (MVD) counts. Rats were euthanized with an overdose of 40 mg/kg intravenous sodium pentobarbital, and then were perfused with 50 mL heparin-saline (50 mg/500 mL) through the ascending aorta, followed by 300 mL formalin-picric solution (4% formaldehyde, 0.4% picric acid in 0.16 mol/L phosphate buffer, pH 7.4). Entire cervical spinal cords were carefully harvested and fixed with 4% phosphate buffer liquid in formaldehyde solution for another 72 h, and the cords were embedded in paraffin.

Microvascular staining and counting: The microvasculature in paraffin-embedded transverse sections (4 μm) were visualized by staining endothelial cells with the CD34 monoclonal antigen using standard immunohistochemical streptavidin-peroxidase staining technique. According to Weidner’s microvascular counting method [24], low power light microscopy (magnification 40×) was used to scan and find three high-density microvascular regions in each section. Individual microvascular counts were conducted on a 400× field. Any brown-stained endothelial cell or endothelial cluster, clearly separate from an immediately adjacent microvascular unit, was considered a single, countable microvascular unit. Neither red blood cells nor vessel lumens were considered necessary for a structure to be defined as a microvascular unit. The MVD were calculated in each section from C3 to C7. The average MVD of three regions was used as the final microvascular count. Assessment of MVD was performed without knowledge of the group and section.

### 4.3. Micro-CT

The spinal cord was subjected to micro-CT scanning after gelatin-lead oxide intracardiac injection. Animals were anesthetized with an overdose of 40 mg/kg of intravenous sodium pentobarbital [22]. An abdominothoracic incision was rapidly performed to expose the heart. An obtuse cannula was inserted into the thoracic aorta via the left ventricle followed by heparinized saline perfusion by adjusting the perfusion height (110 cm H_2_O) and rate (20 mL/min). Subsequently, the right atrium was sheared for use as a venous drain vent. The application of heparinized saline ensured effective removal of the circulatory blood. Effectual perfusion was characterized by effective decoloration of the liver and the presence of limpid drainage fluid. Thereafter, 10% buffered formalin was perfused with the same perfusion height and rate for temporary vessel network fixation. All perfusion solutions were preheated and warmed to 40 °C. Gelatin-lead oxide mixed liquor (medical gelatin 5 g, lead oxide 100 g, distilled water 100 mL) was continually infused into the aortic cannula by syringe (6–8 mL/min) for 5 min, until the contrast liquid that flowed freely from the right atrium vent and the viscera was completely yellow. After the perfusion procedure, the infused animal was moved to a refrigerator for preservation at 4 °C overnight to achieve effective casting of the entire vascular system. Entire cervical spinal cords were then carefully harvested and fixed with 4% phosphate buffer liquid in formaldehyde solution for another 24 h. The muscle tissue was carefully removed from the lamina under a microscope, and the specimens were then subjected to micro-CT scanning (ZKKS-MCT-Sharp-II, Guangzhou ZhongkeKaisheng Medical Technology Co., Ltd., Guangzhou, China). Cross-section, sagittal, and 3D reconstruction figures of each specimen were performed using the 3D-Med 4.3 software analysis system (Guangzhou ZhongkeKaisheng Medical Technology Co., Ltd., Guangzhou, China), and the vascular index (VI) in each segment from C3 to C7 of every specimen was calculated. The VI in the compression and normal levels of the spinal cord were determined for analysis of the correlation between VI and MVD.

### 4.4. Statistical Analysis

Statistical analysis was performed using SPSS 16.0 analysis software (SPSS Inc., Chicago, IL, USA). All measurement results were presented as mean ± standard deviation. MVD values measured in immunohistochemical results from both groups and VI values measured in micro-CT results of both groups were analyzed by the variance test and independent samples *t*-test, while MVD and VI values measured in the compression group were analyzed by correlation and regression analysis; *p* < 0.05 was considered as statistically significant.

## 5. Conclusions

The micro-CT can visualize the microvascular structure in 3D, which can precisely observe the microvascular changes after chronic compression of the spinal cord. In addition, quantitative measurement in 3D micro-CT showed a significant correlation with results from immunohistochemical studies. It suggests micro-CT as an alternative method to provide a sensitive and promising measurement tool for investigating and monitoring microvascular changes in chronic compressive spinal cord injury.
